# Trousseau syndrome with recurrent cerebral infarction as the first oneset in a gastrointestinal malignant tumor patient: A case report

**DOI:** 10.1097/MD.0000000000040146

**Published:** 2024-10-25

**Authors:** Chaoyue Meng, Yuyao Feng, Yi Yang, Kun Zhang, Rui Wang, Ye Wang, Jing Tian, Xiaoyun Liu

**Affiliations:** a Department of Neurology, The First Hospital of Hebei Medical University, Shijiazhuang, Hebei Province, China; b Department of Neurology, The Second Hospital of Hebei Medica University, Shijiazhuang, Hebei Province, China; c Department of Neurology, Beijing Aerospace General Hospital, Beijing, China.

**Keywords:** cerebral infarction, recurrent, Trousseau syndrome, tumor

## Abstract

**Rationale:**

Trousseau syndrome (TS) is a thrombosis disorder characterized by a hypercoagulable state linked to underlying malignancies, resulting in various thrombotic events such as deep vein thrombosis, pulmonary embolism, and arterial thrombosis. This syndrome serves as a crucial indicator of malignancy and can often be the first sign of an underlying tumor. In this case, we report a case of gastrointestinal malignant tumor as the first onset, and analyzes its clinical characteristics to improve the clinicians’ understanding of this kind of disease.

**Patient concerns:**

A 69-year-old woman was admitted to the hospital 4 times in 1 month for cerebral infarction. The patient was admitted several times with a new cerebral infarction lesion and a high D-dimer level, a persistently positive fecal occult blood test, and a gastrointestinal tumor was later found.

**Diagnosis:**

The patient was diagnosed with TS, attributed to her underlying malignancy.

**Interventions:**

During hospitalization, the patients were treated with aspirin for antiplatelet, esomeprazole for protection of gastric mucosa, atorvastatin for lowering blood lipids, butylphthalein for improvement of collateral circulation, edaravone dextrocamphorol for scavenging oxygen free radicals, and betahistine hydrochloride tablets for preventing dizziness.

**Outcomes:**

The patient’s condition improved significantly after initial treatment, but died of the tumor a year after discharge.

**Lessons:**

Currently, TS has a complex and varied clinical presentation and is relatively difficult to diagnose, especially in patients with an unknown tumor history. Focus should be placed on patients with recurrent cerebral infarctions and increased D-dimer levels, and anticoagulation may be an effective treatment for patients with TS.

## 1. Introduction

Trousseau syndrome (TS) is a type of paraneoplastic syndrome, which is characterized by abnormal activation of the blood clotting system in the body, resulting in thromboembolism. Mucin-producing adenomas (e.g., gastric, lung, pancreatic, and ovarian cancers) are more likely to cause TS.^[1,2]^ The diagnosis of TS is based on the presence of multifocal diffusion-restricted lesions on Magnetic Resonance Imaging (MRI) and a hypercoagulable state suggested by elevated D-dimer levels during the acute phase. and hypercoagulability suggested by elevated D-dimer levels in the acute phase. In the case of TS, malignancy activates the patient’s coagulation system, leading to systemic thrombosis and cerebral infarction.^[3,4]^ The incidence of TS is low, the clinical manifestations are diverse, and the current therapeutic regimen continues to follow the principles of anticoagulation with low molecular heparin and symptomatic treatment,^[5]^ but the prognosis is mostly poor. In this article, we report a case of a patient with small bowel malignancy combined with acute cerebral infarction, which may help to improve the accurate understanding of the disease and increase the possibility of early intervention for treatment, thus prolonging patient survival.

## 2. Case details

**Figure 1.**
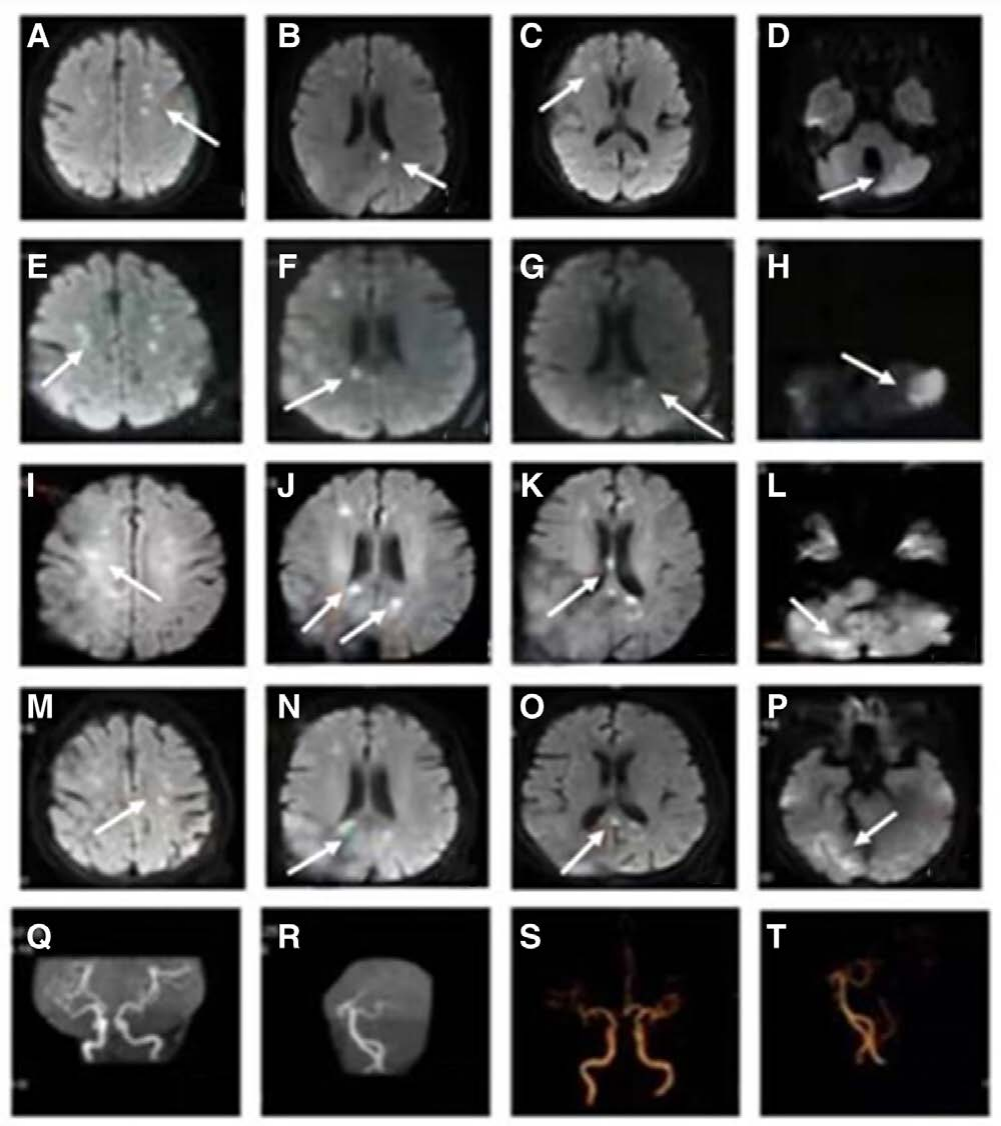
(A–D) MR imaging performance of the patient at the first visit. The arrow in (A) refers to the lesions in bilateral frontal and parietal lobes; The arrow in (B) refers to the left occipital lobe lesion; The arrow in (C) refers to the lesion of the right frontal lobe; The arrow in (D) refers to the left cerebellar lesion; (E–H) Diffusion weighted imaging (DWI) image of the patient at the second onset. (H) shows the new infarction in the left cerebellum. (I–L) DWI after the patient’s first appearance of new symptoms during hospitalization showed new infarcts in the left occipital lobe (J), corpus callosum pressure (K), and bilateral cerebellar hemispheres (L). (M–P) DWI after the second occurrence of new symptoms during hospitalization. A new infarct can be seen in the right occipital lobe at the arrow (P). (Q–T) CTA and MRA accord with arteriosclerosis.

The patient, a 69-year-old woman, was hospitalized with “intermittent dizziness for 2 days.” The patient had a history of hypotension, hepatitis, diabetes, and anemia, and had been hospitalized for acute cerebral infarction half a month earlier (Fig. 1A–D). Physical examination after admission showed: incomplete motor aphasia, normal responsiveness and orientation, no abnormalities in cranial nerve examination, bilateral limb muscle strength level IV, bilateral Babinski sign (−), ataxic movement examination showed poor accuracy of the left finger nose test. Improved auxiliary examination: Brain DWI indicated multiple acute or subacute cerebral infarction (Fig. 1E–H). Magnetic resonance angiography and computed tomography angiography showed no significant abnormalities (Fig. 1Q–T). Abdominal ultrasound showed hepatic cysts. Other positive test results were as follows: Blood routine: red blood cell count 2.72 × 10^12^/L, hemoglobin 74g/L, blood coagulation routine: D-dimer 0.52 µg/mL. Stool routine: fecal occult blood (+). During hospitalization, the patients were treated with aspirin for antiplatelet, esomeprazole for protection of gastric mucosa, atorvastatin for lowering blood lipids, butylphthalein for improvement of collateral circulation, edaravone dextrocamphorol for scavenging oxygen free radicals, and betahistine hydrochloride tablets for preventing dizziness.

**Figure 2.**
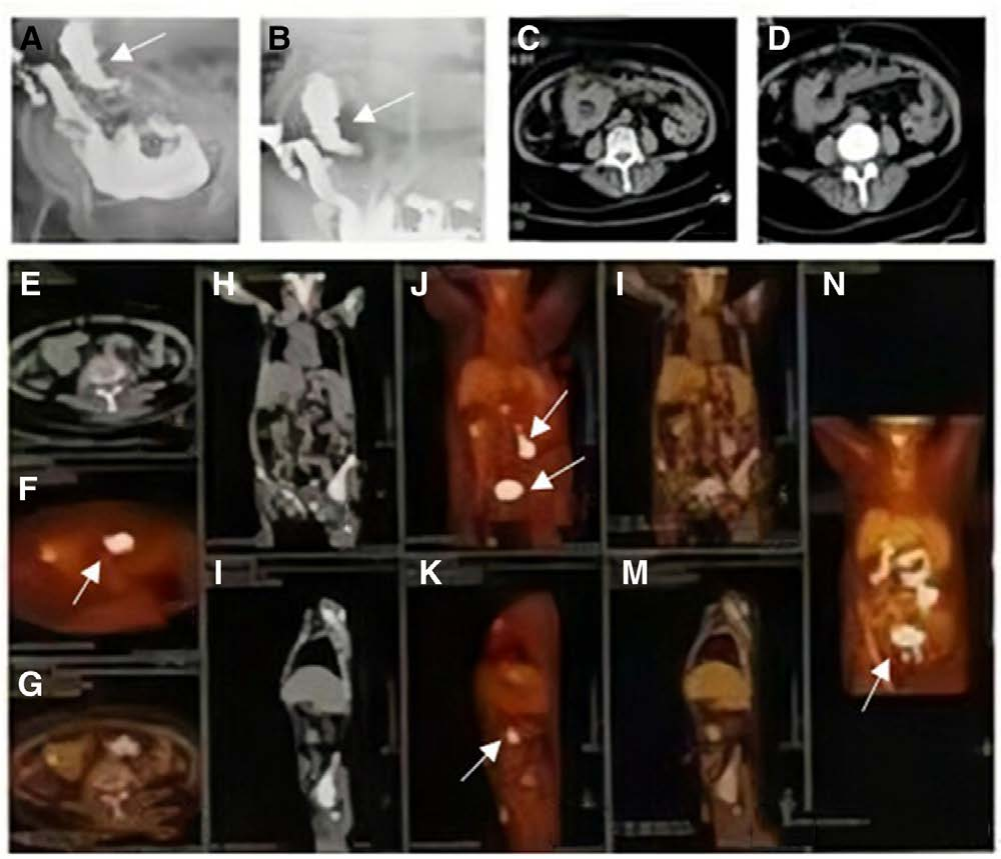
The arrow in (A and B) showed local small intestinal lesions in the right mid-abdomen and abdominal enhanced CT (C and D) of the patient. The arrow in (E–N) showed Abnormal glucose metabolism was observed in the upper abdomen and pelvic small intestine.

On the sixth day of hospitalization, the patient felt weakness in both lower limbs, hallucination occurred several times at night, and reaction force and orientation decreased compared with the previous day. Physical examination showed grade III muscle strength in both lower limbs and bilateral Babinski sign (+). Acute diffusion weighted imaging (DWI) examination of the head revealed multiple acute/subacute infarcts in bilateral frontoparietal temporo-occipital lobe, corpus callosum pressing part, and bilateral cerebellar hemispheres (Fig. 1I–L). Magnetic resonance spectroscopy did not match the tumor profile, so we added low molecular weight heparin (LMWH) sodium anticoagulant therapy. A genetic test was performed on the patient the next day and found the presence of a gene associated with low blood pressure, SERPINA6, but no disease gene associated with recurrent cerebral infarction. 24 hours holter electrocardiogram and transesophageal echocardiography showed no significant abnormalities. Four days later, the patient’s condition worsened again, unable to walk and accompanied by vomiting. DWI examination of the brain showed a new infarction in the right occipital lobe (Fig. 1M–P). Routine blood test results showed hemoglobin 62g/L, and emergency infusion of suspended red blood cells 2U. A bone marrow biopsy and gastrointestinal angiography were performed to determine if the patient had hematological or digestive tract related diseases. Bone marrow examination showed no obvious abnormality. Enterography results showed local small intestinal lesions in the right mid-abdomen (Fig. 2A and B). Enhanced abdominal CT showed diffuse thickening of the right mid-abdomen small intestinal wall and a high possibility of malignant stromal tumor. Simultaneous metastases of the right colon, right liver, and right kidney (Fig. 2C and D). positron emission tomography/computed tomography (PET/CT) was performed on the patient in order to clarify the tumor status of the whole body. The results showed that: Abnormal glucose metabolism was observed in the upper abdomen and pelvic small intestine. Malignant lesions of the small intestine were considered (Fig. 2E–N). Therefore, patients are given anticoagulation with LMWH. After discharge, the patient continued to be treated with oral anticoagulants, nevertheless, which did not prevent the disease from worsening. The patient died half a year later because of multiple organ failure.

## 3. Discussion

TS was first described as migratory superficial thrombophlebitis by Armand Trousseau in 1865. Since Trousseau report is considered to be the first to describe the link between cancer and thrombosis (cancer-associated thrombosis), the combination of cancer and hypercoagulable state has often been referred to as TS since its discovery.^[6]^ Thus TS is a relatively common cancer-related coagulation disorder. The main possibilities for neurological symptoms in combination with malignant tumors are metastasis; paraneoplastic syndrome; and TS. Approximately 7% to 15% of patients with tumors will have symptomatic cerebrovascular disease,^[7]^ but case reports of ischemic stroke as the first manifestation are less common.^[8]^

There are no clear guidelines for the diagnosis of TS-related cerebral infarction. Tsushima^[9]^ et al conducted a retrospective study of 496 patients with acute cerebral infarction. It was found that plasma D-dimer levels at admission were significantly higher in patients with TS than in patients with other causes of cerebral embolism. In addition, female patients, the presence of multiple lesions on DWI, and low platelet and BNP levels were significant predictors of stroke due to TS. This may provide important clinical implications for the diagnosis of whether TS is combined in patients with cerebral embolism. It has been found that in the case of unclear source of embolus or combination of other organic diseases, imaging examination is of some significance in clarifying and differentiating the diagnosis. Multifocal simultaneous high-intensity lesions shown by DWI suggest that the infarct site involves 3 specific regions in the bilateral anterior and posterior circulation, which is clinically referred to as the “three territory sign” (TTS). The presence of TTS suggests that ischemic stroke is highly associated with malignancy.^[10]^ Compared with AF-related acute cerebral infarction, TTS is more frequent in patients with malignancy-related stroke, and TTS is closely associated with the poor prognosis of patients with malignancy-related stroke, which suggests that TTS has diagnostic and prognostic value for malignancy-related hypercoagulable stroke, diagnostic and prognostic value. It provides meaningful clinical ideas for clarifying the diagnosis of occult tumors and preventing delays in the treatment of primary diseases. In this case, the patient was admitted to the hospital with acute cerebral infarction as the first symptom, and after perfecting the relevant tests and examinations, the serum indexes all had different degrees of changes, such as D-dimer values higher than normal, craniocerebral MR + DWI suggesting the possibility of TS, and finally clarifying the diagnosis of malignant tumor of the small bowel, which enabled the active administration of anticoagulant and antitumor treatments, but unfortunately, the patient died of malignant tumor half a year after being discharged from the hospital. The patient in this case presented developed multiple cerebral infarction with blood in the stool and abdominal discomfort. The specific abnormal laboratory marker was the elevation of the D-D polymers; the typical MRI manifestation is the TTS. Finally was diagnosed TS.

Cancer-associated hypercoagulability should be considered an important etiological factor in stroke patients with unexplained disseminated acute cerebral infarction in the absence of conventional stroke risk factors, especially in patients with concomitant multiorgan embolism. Novel oral anticoagulants may be an alternative therapy for long-term control of cancer-related arterial thromboembolism in the setting of effective cancer therapy.^[11]^ The current standard of care for cancer-related venous thromboembolism is anticoagulation with LMWH for at least 3 to 6 months,^[12]^ which has been shown to cause fewer serious bleeding complications than regular heparin,^[13]^ whereas the combined use of heparin and antiplatelet agents increases the risk of bleeding, and should be used with caution. should be used with caution. In patients requiring long-term anticoagulation, LMWH, edoxaban or rivaroxaban for at least 6 months is currently the preferred regimen, and they have better efficacy than vitamin K antagonists (VKA). If LMWH or direct oral anticoagulants (DOAC) are not available, VKA can be used.^[14]^

Currently, there is no accepted diagnostic criterion for TS. when patients are diagnosed, they are usually in an advanced stage, and tumor reduction therapy is difficult to implement. In this case, we focused on the treatment of cerebral infarction, which delayed the diagnosis, and later limited the patient’s own state, which prevented us from completing the pathology and failing to further define the nature of the tumor.

## 4. Conclusion

In summary, TS-induced cerebral embolism may be the first manifestation of cancer, and thus ischemic stroke may be the first manifestation of undiagnosed cancer. TS progresses rapidly and tends to be associated with a poor prognosis, early stroke recurrence and high mortality. If misdiagnosed as other types of stroke, specific treatment strategies for TS may be delayed, thus worsening the condition. Therefore, early diagnostic indicators of malignancy combined with TS derived from the analysis of clinical predictors, active search for the primary cause, timely administration of effective antitumor therapy,^[14]^ and systemic anticoagulation have the potential to improve the outcome of poor prognosis of TS treatment to a certain extent,^[13]^ and prolong the survival time of patients.

## Acknowledgments

I would like to thank all my team members who have helped me to develop the fundamental and essential academic competence.

## Author contributions

**Data curation:** Rui Wang, Ye Wang, Jing Tian.

**Supervision:** Xiaoyun Liu.

**Writing – original draft:** Chaoyue Meng.

**Writing – review & editing:** Yuyao Feng, Yi Yang, Kun Zhang.
